# SerpinB3 Promotes Pro-fibrogenic Responses in Activated Hepatic Stellate Cells

**DOI:** 10.1038/s41598-017-03744-3

**Published:** 2017-06-13

**Authors:** Erica Novo, Gianmarco Villano, Cristian Turato, Stefania Cannito, Claudia Paternostro, Chiara Busletta, Alessandra Biasiolo, Santina Quarta, Elisabetta Morello, Claudia Bocca, Antonella Miglietta, Ezio David, Salvatore Sutti, Mario Plebani, Emanuele Albano, Maurizio Parola, Patrizia Pontisso

**Affiliations:** 1 Department Clinical and Biological Sciences, Unit of Experimental Medicine and Clinical Pathology, University of Torino, Torino, Italy; 2 Department of Medicine, University of Padova, Padova, Italy; 3 Veneto Institute of Oncology IOV – IRCCS, Padua, Italy; 4 Pathology Unit, S. Giovanni Battista Hospital, Torino, Italy; 5 Department of Health Sciences, University “A. Avogadro” of East Piedmont, Novara, Italy

## Abstract

SerpinB3 is a hypoxia- and hypoxia-inducible factor-2α-dependent cystein protease inhibitor that is up-regulated in hepatocellular carcinoma and in parenchymal cells during chronic liver diseases (CLD). SerpinB3 up-regulation in CLD patients has been reported to correlate with the extent of liver fibrosis and the production of transforming growth factor-β1, but the actual role of SerpinB3 in hepatic fibrogenesis is still poorly characterized. In the present study we analyzed the pro-fibrogenic action of SerpinB3 in cell cultures and in two different murine models of liver fibrosis. “*In vitro*” experiments revealed that SerpinB3 addition to either primary cultures of human activated myofibroblast-like hepatic stellate cells (HSC/MFs) or human stellate cell line (LX2 cells) strongly up-regulated the expression of genes involved in fibrogenesis and promoted oriented migration, but not cell proliferation. Chronic liver injury by CCl_4_ administration or by feeding a methionine/choline deficient diet to transgenic mice over-expressing human SerpinB3 in hepatocytes confirmed that SerpinB3 over-expression significantly increased the mRNA levels of pro-fibrogenic genes, collagen deposition and αSMA-positive HSC/MFs as compared to wild-type mice, without affecting parenchymal damage. The present study provides for the first time evidence that hepatocyte release of SerpinB3 during CLD can contribute to liver fibrogenesis by acting on HSC/MFs.

## Introduction

Liver fibrogenesis is a dynamic and highly integrated process that, irrespective of the etiology, results in a progressive accumulation of extracellular matrix (ECM) components and drives the progression of chronic liver disease (CLD)^1–3^. A major pro-fibrogenic role is played by hepatic myofibroblasts (MFs)^1–9^ which mainly originate from a process of activation/trans-differentiation of hepatic stellate cells (HSC) or HSC/MFs^8, 9^. The persistent activation of these cells is the consequence of a complex interaction between growth factors, cytokines, chemokines, reactive oxygen species (ROS) and other mediators^1–7^. In the pro-fibrogenic environment these factors are released by- and interact with- several liver cell populations, including damaged hepatocytes, activated inflammatory cells and MFs^1–7^. Accordingly, the identification and characterization of the mediators involved in sustaining the pro-fibrogenic role of MFs during CLD progression is a critical issue to design novel selective therapeutic strategies and/or to develop diagnostic procedures.

Along these lines, SerpinB3, a member of the family of serine-proteases inhibitors (serpins)^10, 11^, has recently emerged as a mediator involved in CLD progression^12, 13^ and in liver carcinogenesis^14–18^. SerpinB3 expression is regulated by hypoxia through the hypoxia-inducible factor-2α (HIF-2α)^19^. In normal human and murine livers SerpinB3 is virtually undetectable, but its expression is readily appreciable in a significant percentage of liver biopsies from CLD patients, as well as in hepatocellular carcinoma (HCC)^12–18^ and hepatoblastomas^20^. In both neoplastic and non-neoplastic settings, liver SerpinB3 up-regulation correlates with that of transforming growth factor-β1 (TGF-β1), a critical mediator in liver fibrogenesis^12, 21^. In particular, a study performed in liver biopsies from 94 patients with CLD of different etiology has outlined a significant “*in vivo*” correlation between TGF-β1 and SerpinB3 expression (protein and mRNA level). The same study also reported “*in vitro*” data showing that SerpinB3 over-expression in hepatocyte-derived cells also promoted TGF-β1 release, suggesting that SerpinB3 may contribute to up-regulate TGF-β1 production during chronic injury^12^. The putative pro-fibrogenic action of SerpinB3 has also been evidenced in human idiopathic pulmonary fibrosis^22^ and in a murine model of lung fibrosis^23, 24^. Nonetheless, the mechanisms by which SerpinB3 may contribute to liver fibrogenesis are still poorly characterized.

In the present study we investigated the putative pro-fibrogenic role of SerpinB3 first by performing experiments “*in vitro*” using human HSC/MFs and then “*in vivo*” taking advantage of two different experimental protocols of chronic liver injury in transgenic mice overexpressing human SerpinB3 in hepatocytes (TG-SB3 mice).

## Results

### SerpinB3 up-regulates transcription of key pro-fibrogenic genes in human HSC/MFs

**Figure 1:**
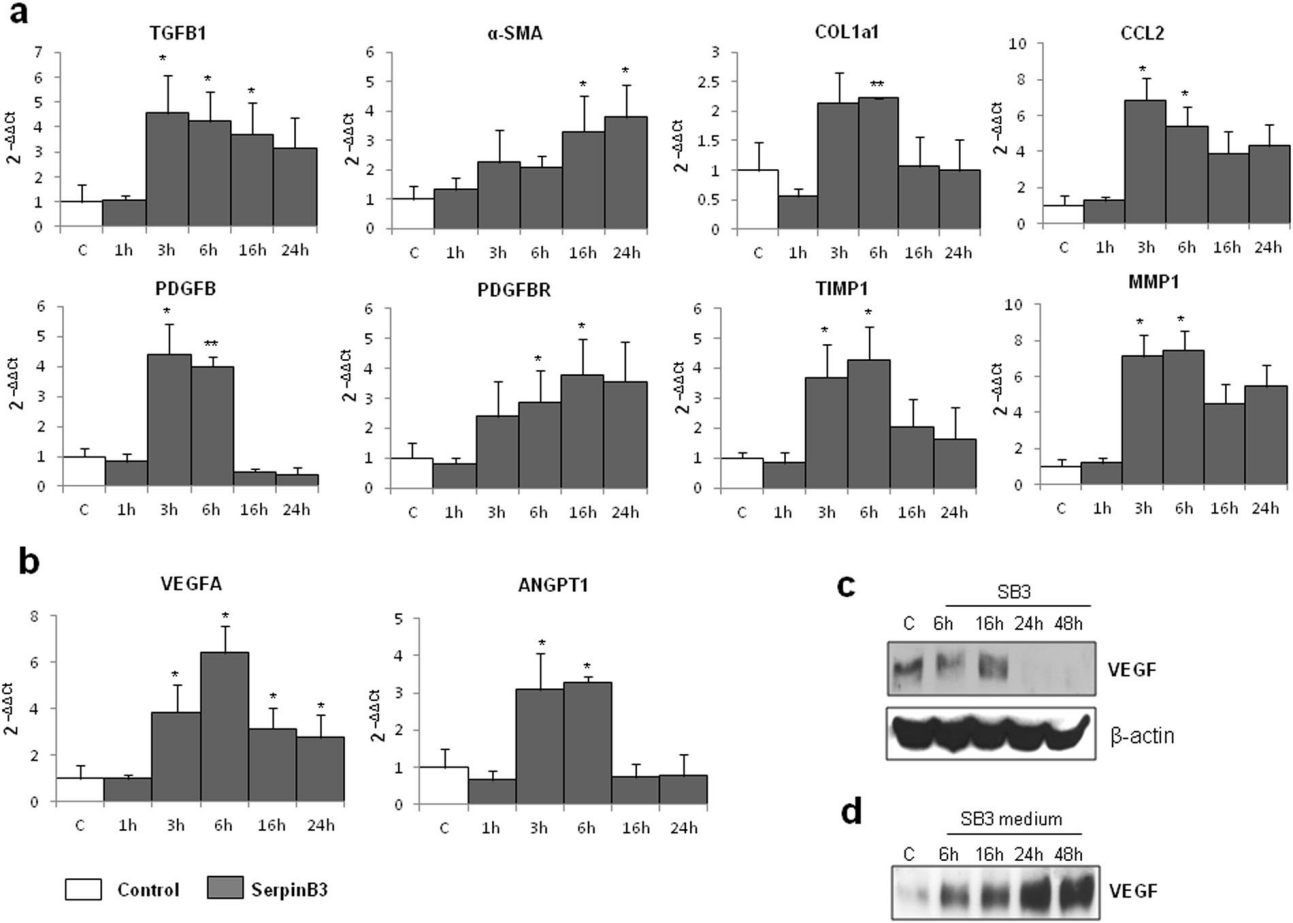
SerpinB3 up-regulates expression of genes involved in liver fibrogenesis in immortalized human HSC (LX2 cells). **(a)** Analysis by quantitative real-time PCR (Q-PCR) of transcript levels of pro-fibrogenic genes, of CCL2 as well as of **(b)** VEGF-A or angiopoietin 1 (Ang-1) in human LX2 cells exposed for the indicated time points to 100 ng/ml human recombinant SerpinB3 (SB3). Data are expressed as means ± SEM of three independent experiments (*p < 0.05 or **p < 0.01 vs control values). **(c,d)** WB Analysis of VEGF-A protein in total extracts (left panel) and immuno-precipitated VEGF-A protein levels released in the extracellular medium collected at the indicated time points. The cropped gels shows in this Fig. have been run under the same experimental conditions.

The addition of human recombinant SerpinB3 (hrSerpinB3) to cultures of human immortalized activated MF-like cells (LX2 cells) resulted in the up-regulation of the transcripts for key pro-fibrogenic genes, including TGFβ1 (TGFB1), COL1A1, α-SMA, platelet-derived growth factor (PDGF)-B and the related PDGF-β receptor (PDGFBR) subunit, TIMP-1, MMP-1 as well as C-C motif chemokine ligand 2 (CCL2) (Fig. 1a). The activation of these genes was also fully reproduced in primary cultures of human HSC/MFs exposed to hrSerpinB3 (Supplementary Fig. 1a). Furthermore, upon hrSerpinB3 addition, both LX2 cells and primary culture of human HSC/MFs up-regulated the transcription of pro-angiogenic cytokines relevant in sustaining liver fibrogenesis^25, 26^ such as VEGF-A (VEGFA) and Angiopoietin-1 (Ang-1 or ANGPT1) (Fig. 1b; Supplementary Fig. 2). In HSC/MFs SerpinB3 also stimulated the expression of TIE-2, the gene encoding for the Ang-1 receptor (Supplementary Fig. 2). Interestingly, already 6 hours after hrSerpinB3 addition LX2 cells released VEGF-A protein in the extracellular medium and this effect reached a maximum in the following 24–48 hours (Fig. 1c,d).

### SerpinB3 induces oriented migration in human HSC/MFs

**Figure 2:**
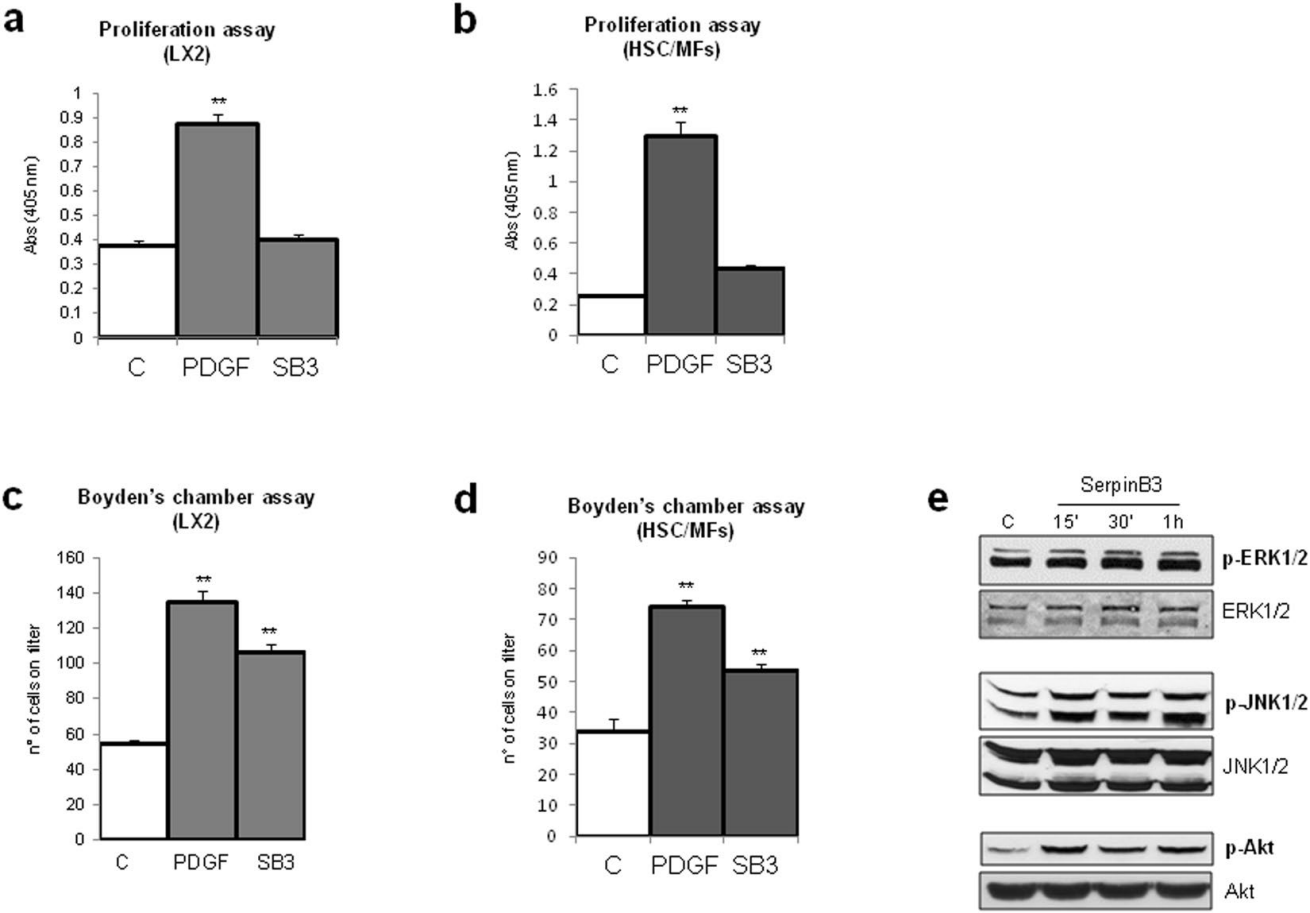
hrSerpinB3 stimulates oriented migration, but not proliferation, in human LX2 cells and human HSC/MFs. **(a,b)** Confluent and 24 hr starved LX2 cells (**a**) or HSC/MFs (**b**) were left untreated or exposed to either hrSerpinB3 (100 ng/ml) or PDGF-BB (10 ng/ml, positive control) for further 48 hrs and then evaluated for proliferation using the crystal violet assay (see Methods). **(c,d)** Analysis of oriented migration in the modified Boyden’s chamber assay of LX2 cells (**c**) or HSC/MFs (**d**) exposed or not to either hrSerpinB3 (100 ng/ml) or PDGF-BB (10 ng/ml, positive control). Data in bar graphs are expressed as means ± SEM of three independent experiments (**p < 0.01 vs control values). **(e)** Western blot analysis of activation of ERK1/2, JNK1/2 and Akt signaling pathways in LX2 cells exposed to hrSerpinB3 (100 ng/ml). Confluent and 24 hr starved LX2 cells were exposed to SerpinB3 for the indicated time points and activation of pathways was evaluated in total extracts by analysis of levels of phosphorylated versus un-phosphorylated levels of ERK1/2, JNK1/2 and c-Akt. The cropped gels shows in this Fig. have been run under the same experimental conditions.

**Figure 3:**
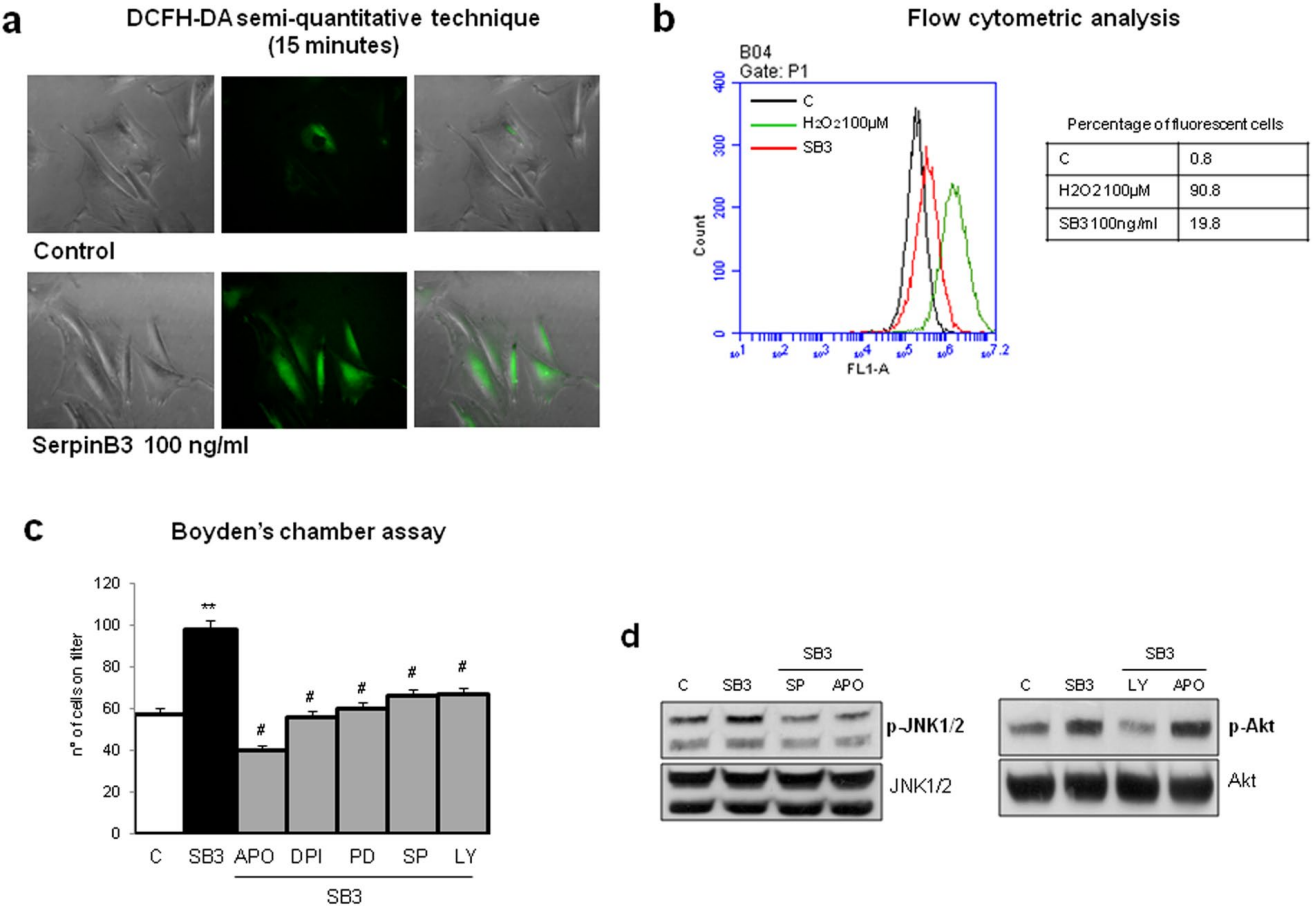
hrSerpinB3-dependent induction of oriented migration relies on intracellular generation of ROS and specific signaling pathways. **(a,b)** Confluent and 24 hr starved LX2 cells were left untreated or exposed to either hrSerpinB3 (100 ng/ml) or PDGF-BB (10 ng/ml, positive control) and then evaluated for early (i.e., 15 minutes) intracellular generation of ROS as shown by DCFH-DA fluorescence (**a**) or flow cytometry analysis (**b**). **(c,d)** Analysis of oriented migration in the modified Boyden’s chamber assay (**c**) or Western blot analysis of activation of JNK1/2 and Akt signaling pathways (**d**) of control LX2 cells or LX2 cells exposed to hrSerpinB3 (100 ng/ml) in the presence of pharmacological inhibitors of JNK1/2 (SP, SP600125,) and Akt (LY, LY294002) or in the presence of apocynin (APO) or diphenylen-iodonium (DPI), all added 30 min before addition of hrSerpinB3. Data in bar graphs are expressed as means ± SEM of three independent experiments (*p < 0.05 or **p < 0.01 vs control values, ^#^p < 0.05 vs related stimulus). Images from Western blot analysis or from evaluation of ROS generation are representative from at least three experiments performed. The cropped gels shows in this Fig. have been run under the same experimental conditions.

We next evaluated whether SerpinB3 may modulate proliferation and chemotaxis of activated HSCs. Human LX2 cells as well as human primary HSC/MFs, which responded positively to the potent mitogen and chemoattractant PDGF-BB (used as positive control), failed to show appreciable increase in proliferation when exposed to hrSerpinB3 (Fig. 2a,b). However, both LX2 cells (Fig. 2c) and human HSC/MFs (Fig. 2d) showed oriented migration when exposed to hrSerpinB3 in a modified Boyden’s chamber assay. Additional experiments, designed to investigate the chemoattractant action, revealed that LX2 cells exposed to hrSerpinB3 had features in common with those induced by other chemo-attractants on activated HSCs^27^. This included very early activation/phosphorylation of c-Jun NH_2_-terminal kinase isoforms −1 and −2 (JNK1/2) and c-Akt, with no relevant change in activation/phosphorylation of extracellular-regulated kinase isoforms 1 and 2 (ERK1/2) (Fig. 2e). In addition, hrSerpinB3 also promoted HSC/MF generation of intracellular ROS that was evident already after 15 min (Fig. 3b). As previously shown for other chemoattractants^27^, hrSerpinB3-induced oriented migration was almost abolished by pre-treating HSC/MFs with pharmacological inhibitors of either NADPH-oxidase (apocynin, Diphenyleneiodonium or DPI) or of JNK1/2 and c-Akt (SP600125 or LY294002) (Fig. 3c). Furthermore, apocynin-mediated inhibition of ROS generation by NADPH-oxidase also blocked the activation/phosphorylation of JNK1/2 but not that of c-Akt (Fig. 3d), confirming that the chemotactic action of SerpinB3 mainly involves the same ROS- and JNK1/2-related signals described for other chemoattractants like PDGF-BB, VEGF-A and CCL2 that are active on HSC/MFs^27^.

### Transgenic mice over-expressing human SerpinB3 in murine hepatocytes develop more liver fibrosis than wild type littermates

**Figure 4:**
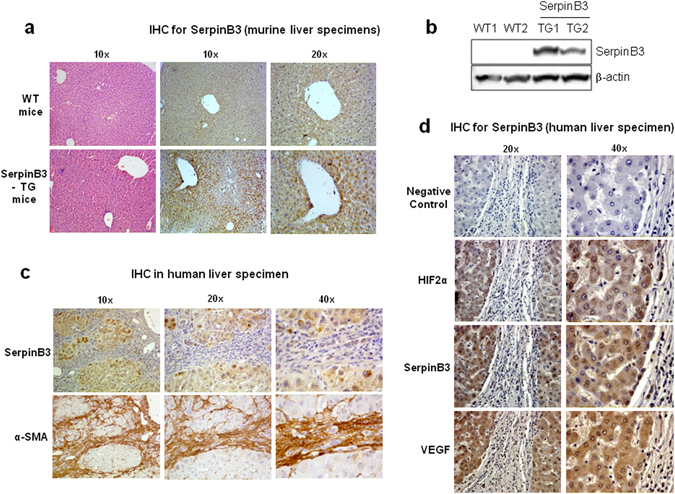
SerpinB3 overexpression in TG mice and human liver specimens from HCV patients. (**a**) Liver morphology (hematoxylin/eosin staining) and immunohistochemistry (IHC) for SerpinB3 expression in the liver of control WT and TG mice. Original magnification as indicated. **(b)** Western blot analysis of SerpinB3 protein levels performed on total liver extracts obtained from either WT and TG mice. Equal loading was monitored by re-blotting membranes for β-actin. The cropped gels shows in this Fig. have been run under the same experimental conditions. Immunohistochemistry analysis for **(c)** SerpinB3 and α-smooth muscle actin (α-SMA) or **(d)** for HIF2α, SerpinB3, and VEGF-A in liver specimens from HCV infected patients (METAVIR F4).

As mentioned above, SerpinB3 is virtually undetectable in either human or murine livers, but it is produced in response to liver hypoxia by hepatocytes or hepatic cancer cells in a significant fraction of patients with cirrhosis or hepatocellular carcinoma^19^. In order to mechanistically investigate whether SerpinB3 may operate as a pro-fibrogenic mediator we employed previously characterized transgenic mice that overexpress human SerpinB3 (TG-SB3 mice) in hepatocytes and in lung epithelial cells^24^. The livers of naïve SerpinB3 TG-mice had normal morphology and hepatocytes of naïve SerpinB3 TG-mice as well as of related wild type mice did not differ for the expression of albumin, cytokeratin 18, α-fetoprotein or the ability to store glycogen and were all negative for cytokeratin 7 expression (Supplementary Figure 3). SerpinB3 expression was evident in parenchymal cells (Fig. 4a) and Western blot analysis confirmed the exclusive presence of human SerpinB3 in TG animals (Fig. 4b). This pattern corresponded to that seen in CLD patients where SerpinB3 immunostaining was mainly detectable in hepatocytes (Fig. 4c). In human specimens from HCV cirrhotic patients (METAVIR F4) SerpinB3-positive cells were usually detectable in hypoxic areas, as shown by the concomitant expression of HIF2α and VEGF-A (Fig. 4d).

**Figure 5:**
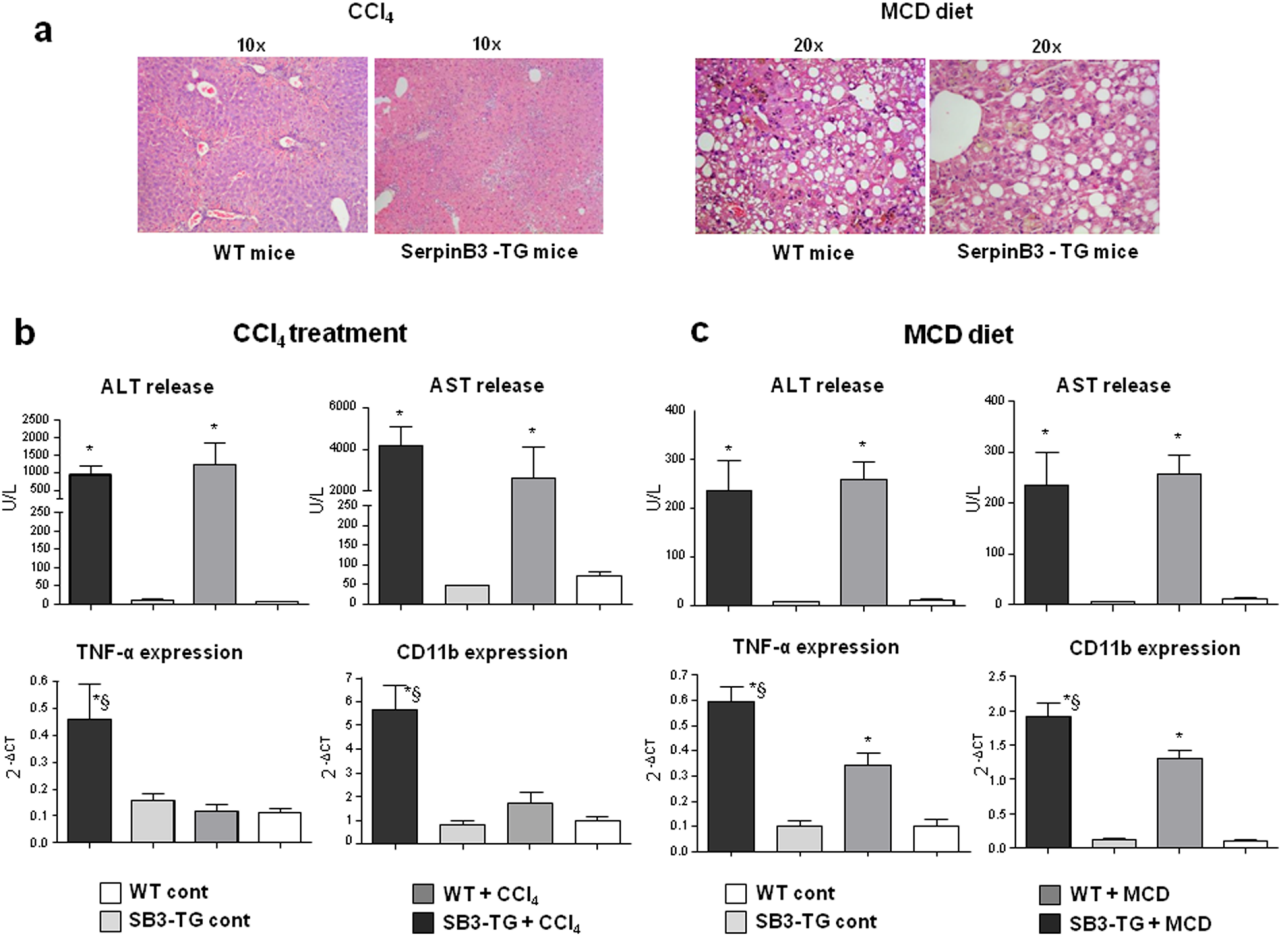
Parenchymal injury and inflammatory markers in SerpinB3 transgenic (SB3-TG) mice and wild type (WT) mice following chronic (10 weeks) CCl_4_ administration or 4 weeks feeding with a methionine/choline deficient (MCD) diet. **(a)** Liver morphology in mice exposed to CCl_4_ or the MCD diet was evaluated by hematoxylin/eosin staining (magnification 10X). Parenchymal injury, estimated by measuring the circulating levels of alanine (ALT) and aspartate (AST) aminotransferases and liver expression of inflammatory markers TNF-α and CD11c, evaluated by quantitative real-time PCR (Q-PCR), are reported in CCl_4_ treated mice **(b)** and in MCD fed mice **(c)**. Data in graphs are expressed as means ± SD (n = 6 mice for each experimental group) (*p < 0.01 vs the relative control mice; ^§^p < 0.05 vs WT-mice receiving CCl_4_ or the MCD diet).

**Figure 6:**
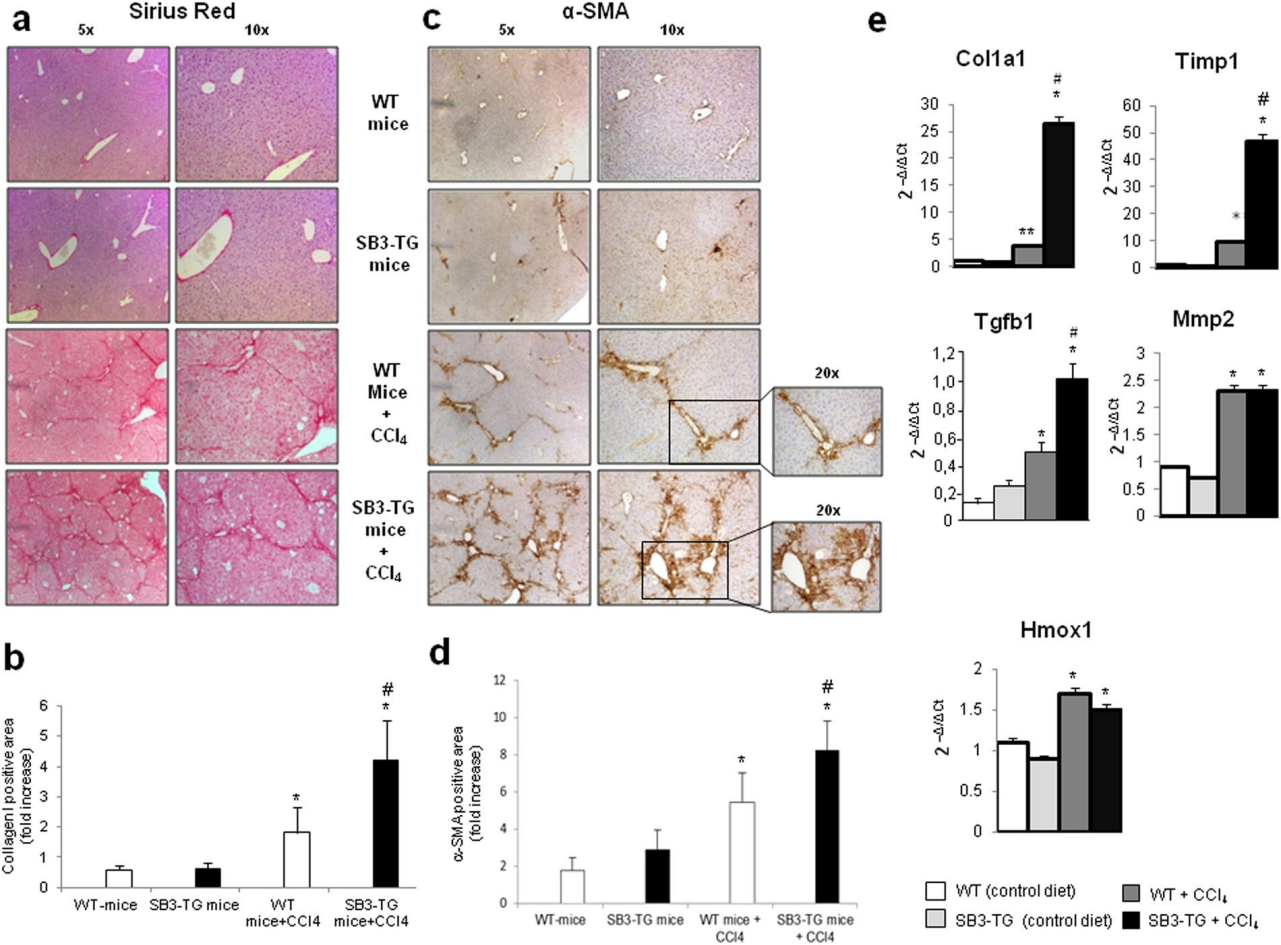
Liver fibrosis “*in vivo*” in SerpinB3 transgenic (TG) mice vs wild type (WT) mice exposed to chronic CCl_4_ administration. **(a,c)** Following chronic exposure to CCl_4_ (10 weeks) liver fibrosis was morphologically evaluated by Sirius red staining (**a**) or by immunohistochemistry for α-SMA (**c**). Original magnification as indicated. **(b,d)** ImageJ software analysis was performed for both Sirius red staining (**b**) and α-SMA immunohistochemistry analysis (**d**) to evaluate the amount of fibrosis. **(e)** Analysis by quantitative real-time PCR (Q-PCR) of SerpinB3 transcript levels of pro-fibrogenic genes in the different experimental groups. Data in graphs are expressed as means ± SEM (n = 6 mice for any experimental group) (*p < 0.05 vs relative control mice; ^#^p < 0.05 vs WT-mice treated with CCl_4_).

**Figure 7:**
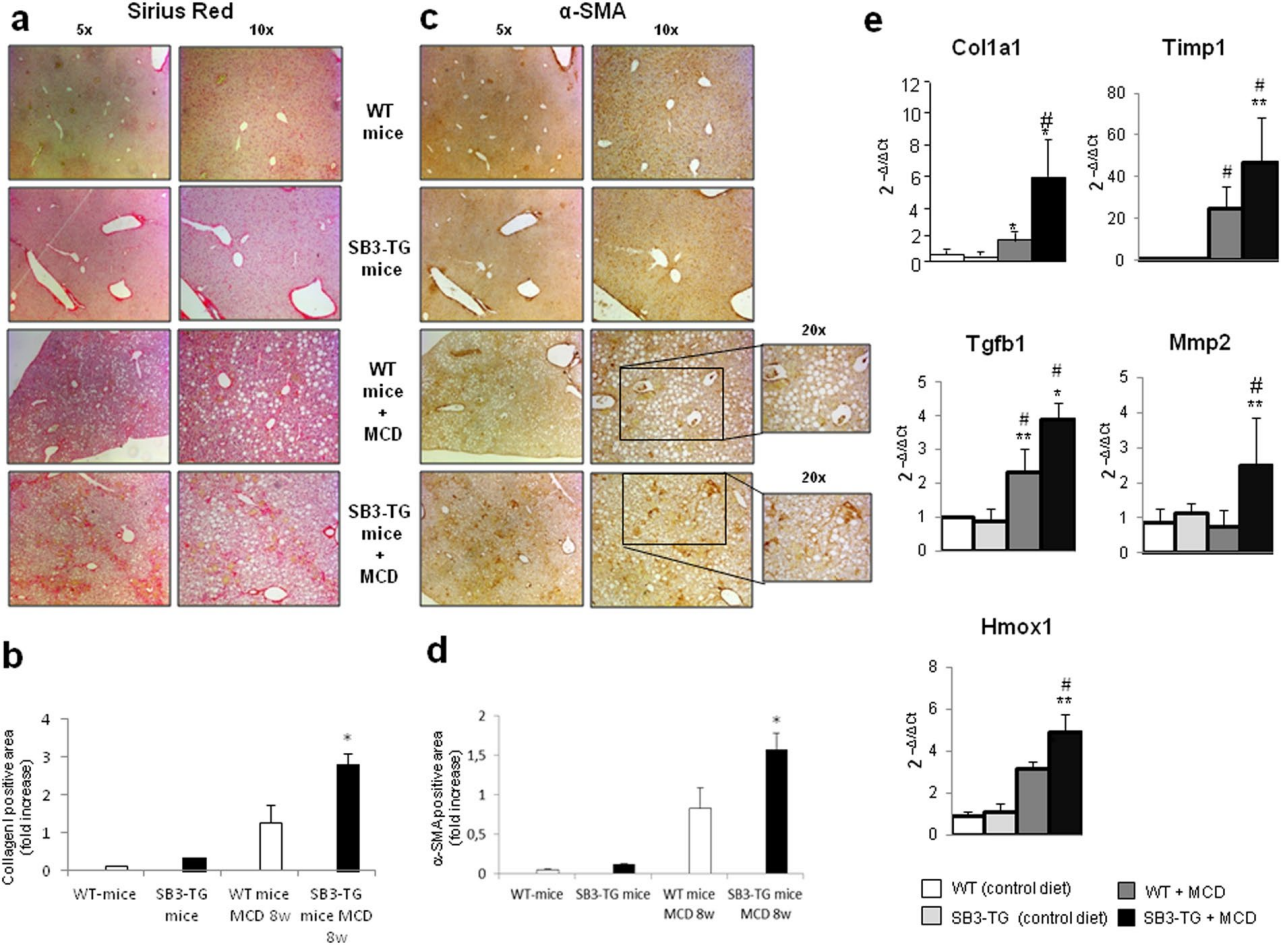
Liver fibrosis “*in vivo*” in SerpinB3 transgenic (SB3-TG) mice vs wild type (WT) mice fed with MCD diet. **(a,c)** Following MCD diet (8 weeks) liver fibrosis was morphologically evaluated by Sirius red staining (**a**) or by immunohistochemistry for α-SMA (**c**). Original magnification as indicated. **(b,d)** ImageJ software analysis was performed for both Sirius red staining (**b**) and α-SMA immunohistochemistry analysis (**d**) to evaluate the amount of fibrosis. (**e**) Analysis by quantitative real-time PCR (Q-PCR) of SerpinB3 transcript levels of pro-fibrogenic genes in the different experimental groups. Data in graphs are expressed as means ± SEM (n = 6 mice for any experimental group) (*p < 0.05 or **p < 0.01 vs relative control mice; ^#^p < 0.05 vs WT-mice fed with MCD diet). Col1a1: collagen 1A1; Timp1: tissue inhibitor of metalloproteases type 1; Tgfb1: TGFβ1; Hmox-1: heme-oxygenase 1; Mmp2: matrix-metalloprotease type 2.

For evaluating the actual impact of SerpinB3 on the evolution of hepatic fibrosis TG-SB3 mice and corresponding WT littermates were exposed to chronic liver injury. To this aim we used two different experimental murine models: (i) chronic CCl_4_ administration, which results in post-necrotic bridging fibrosis resembling pan-lobular/parenchymal fibrosis found in the clinical conditions in which SerpinB3 expression has been best characterized^2, 28, 29^; (ii) mice fed with the MCD diet, that leads to pericellular/perisinusoidal fibrosis similar to that seen in human NAFLD/NASH^2, 28, 29^. Naive TG-SB3 mice had plasma SerpinB3 levels around 2 ng/mL (1.8 ± 0.6 ng/mL) that were enhanced by 5–10 fold in response to chronic liver injury. In particular, CCl_4_-treated animals showed circulating SerpinB3 twice as higher than those receiving the MCD diet (21.5 ± 6.7 ng/mL vs 10.5 ± 5.4 ng/mL; p < 0.001). In the transgenic animals SerpinB3 over-production did not influence hepatocellular damage, as liver histology and the circulating levels of alanine (ALT) and aspartate (AST) aminotransferases were comparable to those in wild type mice (Fig. 5). On the other hand, TG-SB3 mice receiving CCl_4_ or the MCD diet showed a higher hepatic expression of inflammatory markers TNF-α and CD11b (Fig. 5) than the relative wild type animals. In both models of chronic liver injury, collagen Sirius Red staining revealed that the overexpression of SerpinB3 resulted in a marked increase in the extension of fibrosis. In a similar manner the number of α-SMA positive HSC/MFs was significantly higher in TG-SB3 mice exposed to CCl_4_ or the MCD diet than in similarly treated wild-type animals (Figs 6 and 7). Consistently, Q-PCR analysis for a set of genes involved in fibrogenesis demonstrated that in both the experimental models the hepatic transcripts for collagen 1A1 (Col1a1), tissue inhibitor of metalloproteases type 1 (Timp1) and TGFβ1 (Tgfb1) were significantly enhanced in TG-SB3 (Figs 6 and 7). Among TG-SB3 mice receiving CCl_4_ and the MCD diet we also observed a significant positive correlation (r = 0.74; p = 0.04) between the individual levels of circulating SerpinB3 and those of liver collagen 1A1 transcripts. The mRNAs for heme-oxygenase 1 (Hmox-1) and matrix-metalloprotease type 2 (Mmp2) were also elevated in TG-SB3 animals receiving the MCD diet, but not in those exposed to CCl_4_ (Figs 6 and 7). Altogether, these data provide *in vitro* and *in vivo* evidence supporting the implication of SerpinB3 in the processes leading to CLD progression to fibrosis.

## Discussion

SerpinB3 is virtually undetectable in normal human livers, but several studies have evidenced the presence of SerpinB3 in liver biopsies from patients with CLD, mostly chronic HCV infection, where it has been associated ﻿to CLD progression and liver carcinogenesis^13–21^. In fact, in CLD patients SerpinB3 up-regulation correlates with TGF-β1 expression and the extent of hepatic fibrosis^12^. Furthermore, experiments using hepatocyte-derived cell lines over-expressing SerpinB3 have evidenced its role in promoting TGF-β1 production, suggesting SerpinB3 as a putative pro-fibrogenic mediator. More recently, SerpinB3 expression has been reported to be up-regulated by hypoxia through a HIF-2α-dependent and ROS-modulated mechanism^19^. This is a potentially critical issue, since in the last decade liver hypoxia and angiogenesis have been suggested to parallel or even drive the fibrogenic progression of CLDs^6, 25, 26, 29–31^. Literature data relating SerpinB3 with advanced cirrhosis and hepatocellular carcinoma have also evidenced that, beside enhancing TGF-β1 expression, SerpinB3 can induce epithelial-mesenchymal transition (EMT) of hepatoma cancer cells, resulting in their increased invasiveness and proliferation^12, 17, 19, 21^. Indeed, in HCC patients high levels of SerpinB3 were significantly associated with early tumour recurrence and poor prognosis^21^. However, the involvement of EMT in liver fibrogenesis has been recently challenged, with most researchers suggesting a minor, if any, pro-fibrogenic role for EMT and supporting the concept that MFs originate mainly from activation/trans-differentiation of HSC^4, 6, 8, 9, 32^. These considerations have led us to explore the hypothesis that SerpinB3 might directly affect the behaviour of MF-like cells.

Our *in vitro* experiments show that hrSerpinB3 can directly act on activated HSCs by both up-regulating the transcription of critical genes involved in fibrogenesis and stimulating their oriented migration. In these settings, the capacity of the cells to readily respond to hrSerpinB suggests that SerpinB3 may exert its action on a receptor-mediated basis. Furthermore, at difference of other mediators involved in fibrogenesis, SerpinB3 action on human activated HSCs seems quite selective and does not involves cell proliferation. Unfortunately, at present a specific receptor for SerpinB3 has not yet been characterized. Whatever might be the receptor involved, in human HSC/MFs SerpinB3 promotes NADPH-oxidase-dependent generation of intracellular ROS and ROS-related activation of JNK1/2 signalling pathways. These intracellular events have been implicated in both up-regulation of ECM synthesis/remodelling by MFs as well as in the induction of their oriented migration^1–6^. The latter is a relevant issue for these cells in order to align with nascent and more mature fibrotic septa. On this respect, it is noteworthy that the signal pathways leading to SerpinB3-induced oriented migration are identical to those characterized in human HSC/MFs migrating in response to chemo-attractant polypeptides (PDGF-BB, CCL2 and VEGF) or hypoxic conditions^27, 30^.

Beside the chemotactic action, the exposure of HSC/MFs or LX2 cells to hrSerpinB3 also results in an increased transcription of genes relevant for the fibrogenic progression of CLD along with VEGF-A, Angiopoietin-1 and CCL2, suggesting that SerpinB3 may combine pro-fibrogenic and pro-angiogenic activities. Of interest, the pro-fibrogenic genes stimulated “*in vitro*” by hrSerpinB3 are also up-regulated in response to chronic liver injury in transgenic mice over-expressing human SerpinB3. Accordingly, by using SerpinB3 transgenic mice we observed that the induction of chronic liver injury by repeated exposure to CCl_4_, or the development of steatohepatitis in mice fed the MCD diet leads to a massive stimulation in SerpinB3 production by the hepatocytes and a very significant increase in ECM deposition. This later effect was associated with an increase in the number of α-SMA-positive cells (i.e., MF-like cells) as well as in an enhanced transcription of several pro-fibrogenic genes, including collagen 1A1, TGFβ1, α-SMA, PDGF-B and TIMP1 as compared to WT-mice. This scenario resembles that previously outlined for bleomycin-induced lung fibrosis, in which a direct correlation between SerpinB3 expression, TGFβ1 levels and lung fibrosis was reported^24^. The enhanced deposition of extracellular matrix observed in SerpinB3 transgenic mice exposed to chronic liver injury is independent from the extent of parenchymal injury supporting *in vitro* data concerning direct stimulation of MF-like cells by SerpinB3. Nonetheless, we have observed that hepatic damage in SerpinB3 transgenic mice associates with an enhanced expression of inflammatory markers. At present, it is still unclear whether SerpinB3 can directly sustain the activation of liver inflammatory cells but since chronic inflammation is one of the driving force for the progression of hepatic fibrosis, we cannot exclude that a pro-inflammatory action of SerpinB3 might also contribute to the dramatic increase in liver collagen deposition observed in our experimental settings. By contrast, we do not have *in vitro* or *in vivo* evidence that other non-parenchymal cells, in particular macrophages, may express and release SerpinB3.

A previous study has shown that SerpinB3 expression in the liver is modulated by hypoxia through HIF2α-dependent mechanisms^19^. Based on the knowledge that hypoxic conditions have a pro-fibrogenic and pro-angiogenic role during the progression of CLD^1–6, 25, 26, 29–34^, our study points on the possible implication of SerpinB3 among the mediators responsible for the pro-fibrogenic action of hepatic hypoxia.

In conclusion, the present study provides for the first time evidence indicating that SerpinB3 produced by liver parenchymal cells can contribute to the fibrogenic progression of CLDs by stimulating the responses of HSC/MFs.

## Materials and Methods

### ***In vitro*** experiments with activated, MF-like, hepatic stellate cells or HepG2 cells

Human LX2 cells, a model of immortalized and activated, MF-like, human HSC, were kindly provided by Prof. Scott L. Friedman and were cultured in Dulbecco’s modified Eagle’s medium (Sigma Aldrich Spa, Milan, Italy), supplemented with 10% fetal calf serum and 1% antibiotics. In some experiments we also used human HSC that were isolated from surgical wedge sections of at least three different human livers not suitable for transplantation as previously described^35^. These HSCs, kindly provided by Prof. Fabio Marra, were cultured as previously described^27^ and used between passages 4 and 7 when showing a phenotype of fully activated, MF-like HSCs (HSC/MFs), plated to obtain the desired sub-confluence level and then left for 24 hrs in serum-free Iscove’s medium to have cells at the lowest level of spontaneous proliferation. In *in vitro* experiments LX2 cells or HSC/MFs were exposed to human recombinant SerpinB3 (hrSerpinB3, 100 ng/ml), obtained as previously described^17^.

### Detection of intracellular generation of ROS

Detection of ROS generation in cultured cells was performed by either the conversion of 2′,7′-dichlorodihydrofluorescein diacetate (DCFH-DA, 1 μM) into the corresponding fluorescent derivative or by combining DCFH-DA technique and flow cytometric analysis as previously described^19, 27^. More details are available in the Supplementary Material section.

### Quantitative real-time PCR (Q-PCR)

RNA extraction, complementary DNA synthesis, quantitative real-time PCR (Q-PCR) reactions were performed as previously described^19, 21^. mRNA levels were measured by Q-PCR, using the SYBR® green method as described^21^. More details and oligonucleotide sequences of primers used for Q-PCR are available in the Supplementary Material section.

### Proliferation and Chemotaxis

Proliferation of human LX2 cells or primary HSC/MFs was evaluated by crystal violet proliferation assay by seeding cells in a 96-well plate at a density of 1.5 × 10^3^ cells per well. The cells were incubated in serum-free medium (SFM) for 24 hrs at 100 ng/ml hrSerpinB3 concentration. At the desired time, the medium was removed, and the cells were washed twice with phosphate-buffered saline, fixed with 11% glutaraldehyde; after fixation, cells were washed with H_2_O bd and then stained with 0.1% (w/v) crystal violet solution for 10 min. After washing with water, the crystal violet was solubilized with 50 μl of 10% acetic acid solution, and absorbance was measured at 595–650 nm using a microplate reader (SpectraMAX M3; Molecular Devices, Sunnyvale, CA, USA). Chemotaxis of human LX2 cells or HSC/MFs was evaluated by performing the modified Boyden’s chamber assay as previously described^27, 30^.

### Experimental fibrosis in SerpinB3 transgenic mice and related wild type mice

The two protocols for experimental fibrosis employed in this study were performed by taking advantage of a model of C57Bl/6 transgenic (TG) mice, fully characterized in previous studies^23, 24^, that overexpress human SerpinB3 in the liver and lungs. Full details of the experimental protocols on TG-mice and age-matched C57Bl/6 wild type (WT) mice, receiving either i) chronic administration of carbon tetrachloride (CCl_4_, Sigma-Aldrich, Milano, Italy) for 10 weeks as described by Wang *et al*.^36^ or ii) the methionine- and choline-deficient (MCD) diet up to 8 weeks (as a model of NAFLD/NASH-related fibrosis)^37^ are described in the Supplementary Material section. Experiments and protocols, approved by the Animal Ethical Committee of University of Padua and by the Animal Investigation Committee of the Italian Ministry of Health, were performed in accordance with the Helsinki convention and national guidelines and regulations for animal experiments provided by Italian Ministry of Health.

### Serological analysis

ALT and AST were determined in serum by laboratory routine assays. Circulating SerpinB3 was evaluated by enzyme-linked immunozymatic assay (SCCA-ELISA, Xeptagen, Marghera, VE, Italy) using biotinylated rabbit anti-human SerpinB3 antibody as previously described^15^.

### Immunohistochemistry, Sirius Red staining and histo-morphometric analysis

Paraffin liver sections of cirrhotic specimens derived from patients with HCV-related liver cirrhosis (METAVIR F4) were employed. The use of human material conforms to the ethical guidelines of the 1975 Declaration of Helsinki and was approved for this study by the University of Torino Bioethical Committee. Immunostaining procedure^19, 27^ and Sirius Red staining^38^ were as previously described, as detailed in the Supplementary Material section. Quantification of fibrosis in the murine liver was performed by histo-morphometric analysis using a digital camera and a bright field microscope to collect images that were then analyzed by employing the ImageJ software.

### Statistical analysis

The data presented are means ± SEM or SD and were obtained from at least three independent experiments. Luminograms and morphological images are representative of at least three experiments with similar results. Statistical analysis for these experiments was performed by Student’s t-test or one way ANOVA with Kruskal-Wallis correction for multiple analysis. Significance was taken at p < 0.05.

## Electronic supplementary material

Supplementary Information
