# Supplementary material for: SerpinB3 Promotes Pro-fibrogenic Responses in Activated Hepatic Stellate Cells
Source: Sci Rep. 2017 Jun 13;7:3420. doi: 10.1038/s41598-017-03744-3 (PMC5469760; doi:10.1038/s41598-017-03744-3)
Supplement: Supplementary file 1 — Supplementary Information [file 41598_2017_3744_MOESM1_ESM.doc]

**Supplementary information**

**Manuscript: SerpinB3 Promotes Pro-fibrogenic Responses in Activated Hepatic Stellate Cells**

By: **Erica Novo**^1†^, **Gianmarco Villano**^2†^, Cristian Turato^3^, Stefania Cannito^1^, Claudia Paternostro^1^, Chiara Busletta^1^, Alessandra Biasiolo^2^, Santina Quarta^2^, Elisabetta Morello^1^, Claudia Bocca^1^, Antonella Miglietta^1^, Ezio David^4^, Salvatore Sutti^5^, Mario Plebani^2^, Emanuele Albano^5^, Maurizio Parola^1^*, Patrizia Pontisso^2^.

^1^Dept. Clinical and Biological Sciences, Unit of Experimental Medicine & Clinical Pathology, University of Torino, Italy; ^2^ Dept. Medicine, University of Padova, Italy; ^3^ Veneto Institute of Oncology IOV – IRCCS, Padua, Italy;  ^4^Pathology Unit, Ospedale S. Giovanni Battista, Torino, Italy; ^5^Dept. of Health Sciences, University“Amedeo Avogadro”of East Piedmont, Novara, Italy.

**Supplementary Materials and Methods**

**Materials**

Enhanced chemiluminescence (ECL) reagents, nitrocellulose membranes (Hybond-C extra) were from Amersham Pharmacia Biotech Inc. (Piscataway, NJ, USA). Monoclonal antibody against SerpinB3 (sc-21767), p-ERK1/2 (sc-7383) polyclonal antibody for ERK1/2 (sc-292838), p-Akt1/2/3 (sc-7985-R), Akt1/2/3 (sc-8312), VEGF (sc-152) and HIF-1α (sc-10790) were from Santa Cruz Biotechnology (Santa Cruz, CA, USA). Polyclonal antibody for p-JNK and JNK1/2 were from Cell Signaling Technology (Massachusetts, USA) and polyclonal antibody for HIF-2α was from Novus Biologicals (Cambridge, UK). SP600125 and LY294002 were from Calbiochem (La Jolla, CA, USA). Monoclonal antibodies for β-actin and all other reagents of analytical grade were from Sigma Chemical Co (Sigma Aldrich Spa, Milan, Italy), Boyden’s chambers were from Neuro Probe, Inc. (MD, USA). Human recombinant SerpinB3 (hrSerpinB3) was produced in our laboratory, as previously described [1].

**Experimental fibrosis in SerpinB3 transgenic mice**

The two protocols for experimental fibrosis were carried out by taking advantage of the murine model of C57BL/6 transgenic mice (TG) that overexpress in the liver and lungs human SerpinB3, a transgenic mouse model which has been fully characterized in a previous study [2]. Each of the experimental protocol was performed on 8 TG mice 12-14 weeks old, as well as on an equal number of age-matched C57BL/6 wild type (WT) mice that were used as controls. Mice were submitted then to the following two protocols of chronic liver injury.

1. Chronic carbon tetrachloride (CCl4) administration. Protocol of chronic liver injury as described by Wang et al. [3] requiring chronic administration of the hepatotoxin carbon tetrachloride (CCl_4_, Sigma-Aldrich, Milano, Italy) for 10 weeks. Briefly, mice received intraperitoneally a CCl_4_ dose of 50 µl/100 g body weight, dissolved in olive oil (20%), twice a week for 10 weeks [3]. The control group (6 TG and 6 WT) received injections of olive oil vehicle twice a week for 10 weeks. All mice were sacrificed 24 h after the last dose of treatment.

2. Methionine - choline deficient diet (MCD dietary model). Protocol of chronic liver injury reproducing non-alcoholic fatty liver disease (NAFLD) progressing to non-alcoholic steatohepatitis (NASH) and fibrosis [4]. Briefly, mice were fed for 8 weeks with the MCD diet or control diet (Laboratori Dottori Piccioni, Gessate, Italy) as previously described [5].

In all murine experiments, TG and WT mice were kept under specific pathogen-free conditions and maintained with free access to pellet food and water at the Animal Care Facility of the Experimental Surgery Center, University of Padua, Italy. Liver samples were obtained and either immediately used/processed for molecular biology and morphological purposes or immediately frozen and thereafter maintained at -80°C for further analyses.

**Detection of intracellular generation of ROS**

Cultured cells, seeded in 12-well culture plates (10^5^ cells/well), were either exposed to hrSerpinB3 100ng/ml or treated or not with 100μM H_2_O_2_, used as positive control, for 15 minutes. ROS generation was detected as the conversion of 2’,7’-dichlorodihydrofluorescein diacetate (DCFH-DA, 1 μM) into the corresponding fluorescent derivative. Cells were observed and photographed under a Zeiss fluorescence microscope as previously described [6,7]. ROS generation was also detected by combining DCFH-DA technique and flow cytometric analysis [6]. Cells were seeded in P35 dishes (5x10^5^ cells/dish), cultured for 24 hrs and treated with DCFH-DA 1 μM for 15 minutes to 37°C and then exposed to 100 ng/ml hrSerpinB3 or 100 μM H2O2 for 15 minutes. Cells were rapidly washed with PBS, collected by trypsinization and re-suspended in PBS for analysis. Detection of DCF green fluorescence (FL1) was performed on at least 5,000 cells per sample with a FACScan equipped with a 488 nm argon laser using the CellQuest software (BectonDickinson, Milano, Italy). The peak of FL1 intensity of DCFH-DA-stained control cells was set to channel 101 and retained for all measurements.

**Immunohistochemistry, Sirius Red staining and other stainings**

Immunostaining procedure was as previously described [6,8]. Briefly, paraffin sections (2 μm thick), mounted on poly-L-lysine coated slides, were incubated with the following antibodies: monoclonal antibody against SerpinB3 (Santa Cruz Biotechnology, CA, USA; dilution 1:50), monoclonal antibody against α-SMA (Sigma Aldrich Spa, Milan, Italy), polyclonal antibodies (AbCam, Cambridge, UK) against cytokeratin 18 (dilution 1:800), cytokeratin 7 (dil. 1:8000) or murine albumin (dil. 1:200) or polyclonal antibody against α-fetoprotein (DAKO, Glostrup, Denmark; dil. 1:800). After blocking endogenous peroxidase activity with 3% hydrogen peroxide and performing microwave antigen retrieval, primary antibodies were labeled by using EnVision, HRP-labeled System (DAKO, Glostrup, Denmark) and visualized by 3’-diaminobenzidine substrate. For negative controls the primary antibodies were replaced by isotype- and concentrations-matched irrelevant antibody. Sirius Red stainings were performed by a slight modification of the procedure previously described [9] consisting in a rapid immersion of slides in diluted hematoxylin in order to obtain nuclear counterstain of liver sections. Ten randomly selected fields (x10 magnification) of picrosirius red-stained liver sections (3-4 µm thick) were analysed in three sections of each animal at a final magnification of x100. Quantification of fibrosis was performed by histomorphometric analysis using a digital camera and a bright field microscope to collect images that were then analysed by employing the ImageJ software. Conventional histological stainings (Hematoxylin and Eosin, PAS stain for glycogen) were performed on paraffin sections (2 μm thick).

**Western Blot analysis**

Total cell lysates or nuclear vs cytosolic extracts, obtained as described [6,7], were subjected to sodium dodecyl sulfate-polyacrylamide gel-electrophoresis on 12%, 10% or 7.5% acrylamide gels, incubated with desired primary antibodies, then with peroxidase-conjugated anti-mouse or anti-rabbit immunoglobulins in Tris-buffered saline-Tween containing 2% (w/v) non-fat dry milk and finally developed with the ECL reagents according to manufacturer’s instructions. Sample loading was evaluated by reblotting the same membrane with the un-phosphorylated form of protein or with β-actin antibody.

**Quantitative real-time PCR (Q-PCR)**

RNA extraction, complementary DNA synthesis, quantitative real-time PCR (Q-PCR) reactions were performed as previously described [6,10]. mRNA levels were measured by Q-PCR, using the SYBR® green method as described [10]. The amplification mix was prepared using iTaq Universal Syber Green SuperMix (Biorad Laboratories, Berkeley, CA) following manufacturer’s instructions and realtime PCR was performed using Miniopticon ThermoCycler Instrument (Biorad Laboratories, Berkeley, CA). Oligonucleotide sequence of primers used for RT-PCR were:

***a) Mouse genes***:

Col1a1 sense: 5’-GGGCAAGACAGTCATCGAAT, reverse: GGTGGAGGGAGTTTACACGA; Tgfb1 sense: TTGCTTCAGCTCCACAGAGA, reverse: TGGTTGTAGAGGGCAAGGAC; Pdgfa sense: ACCAGGACGGTCATTTACGA, reverse: GGTGTTACAACAGCCAGTGC; Pdgfb sense: CCAGATCTCTCGGAACCTCA, reverse: GGCTTCTTTCGCACAATCTC; Timp1 sense: ATGCCCACAAGTCCCAGAAC, reverse: TACGCCAGGGAACCAAGAAG; Mmp2 sense: CCAACTACAACTTCTTCCCCC, reverse: CGAGCAAAAGCATCATCCAC; Hmox1 sense: CACGCATATACCCGCTACCT, reverse: AAGGCGGTCTTAGCCTCTTC;

***b) Human genes:***

TGFB1 sense: GGGACTATCCACCTGCAAGA, reverse: CCTCCTTGGCGTAGTAGTCG; α-SMA sense: ACCCACAATGTCCCCATCTA, reverse: GAAGGAATAGCCACGCTCAG; COL1A1 sense: GTGCTAAAGGTGCCAATGGT, reverse: ACCAGGTTCACCGCTGTTAC; CCL2 sense: CCCCAGTCACCTGCTGTTAT, reverse: AGATCTCCTTGGCCACAATG; PDGFB sense: TCCCGAGGAGCTTTATGAGA, reverse: GGGTCATGTTCAGGTCCAAC; PDGFBR sense: GGTGACACTGCACGAGAAGA, reverse: CAATGGTGGTTTTGCAGATG; TIMP1 sense: AATTCCGACCTCGTCATCAG, reverse: TGCAGTTTTCCAGCAATGAG; MMP1 sense: AGGTCTCTGAGGGTCAAGCA, reverse: CTGGTTGAAAAGCATGAGCA; HMOX1 sense: ATGACACCAAGGACCAGAGC reverse: GTGTAAGGACCCATCGGAGA.

Gliceraldehyde-3-phosphate dehydrogenase (GAPDH) was used as internal reference and co-amplified with target samples using identical Q-PCR conditions. Samples were run in triplicate and mRNA expression was generated for each sample. Specificity of the amplified PCR products was determined by melting curve analysis and confirmed by agarose gel electrophoresis.

In order to evaluate murine transcripts for TNFα, CD11b and β-actin liver RNA was retro-transcribed with High Capacity cDNA Reverse Transcription Kit (Applied Biosystems Italia, Monza, Italy). RT-PCR for these transcripts was performed in a Techne TC-312 thermacycler (TecneInc, Burlington NJ, USA) using TaqMan Gene Expression Master Mix and TaqMan Gene Expression probes for indicated murine genes (Applied Biosystems Italia, Monza, Italy) as previously described by some of us (5).

**References for Supplementary Materials and Methods**

1. Turato, C., *et al.* SERPINB3 modulates TGF-β expression in chronic liver disease. Lab Invest 90, 1016-1023 (2010).

2. Lunardi, F., *et al.* Overexpression of SERPIN B3 promotes epithelial proliferation and lung fibrosis in mice. Lab Invest 91**,** 945-954 (2011).

3. Wang, L., *et al.* Effects of retinoic acid on the development of liver fibrosis produced by carbon tetrachloride in mice. Biochimica et Biophysica Acta 1772**,** 66-71 (2007).

4. Hebbard, S. & George, J. Animal models of nonalcoholic fatty liver disease. Nat Rev Gastroenterol Hepatol 8**,** 35-44 (2011).

5. Locatelli, I., *et al.* Endogenous annexin A1 is a novel protective determinant in nonalcoholic steatohepatitis in mice. Hepatology 60**,** 531-544 (2014).

6. Cannito, S., *et al.* Hypoxia up-regulates SERPINB3 through HIF-2α in liver cancer cells. Oncotarget 6, 2206-2221 (2015).

7. Novo, E., *et al.* The biphasic nature of hypoxia-induced directional migration of activated human hepatic stellate cells. J Pathol 226, 588-597 (2012).

8. Novo, E., *et al.* Intracellular reactive oxygen species are required for directional migration of resident and bone marrow-derived hepatic pro-fibrogenic cells. J Hepatol 54, 964-974 (2011).

9. Galastri, S., *et al.* Lack of CC chemokine ligand 2 differentially affects inflammation and fibrosis according to the genetic background in a murine model of steatohepatitis. Clin Sci 123, 459-471 (2012).

10. Turato, C., *et al.* SERPINB3 is associated with TGF-β1 and cytoplasmic β-catenin expression in hepatocellular carcinomas with poor prognosis. Br J Cancer **110**, 2708-2715 (2014).

**Supplementary Figures**

**Legend of Supplementary Figures**

**Supplementary Figure S 1.** SerpinB3 up-regulates transcription of critical genes involved in fibrogenesis or inflammation in cultures of human HSC/MFs. Analysis by quantitative real-time PCR (Q-PCR) of transcript levels of the indicated pro-fibrogenic genes as well as of MCP-1 or CCL2 in human HSC/MFs exposed for the indicated time points to 100ng/ml human recombinant SerpinB3 (SB3). Data are expressed as means ± SEM of three independent experiments (*p< 0.05 or **p< 0.01 vs control values).

**Supplementary Figure S 2.** hrSerpinB3 up-regulates transcription of critical genes involved in angiogenesis in cultures of human HSC/MFs. Analysis by quantitative real-time PCR (Q-PCR) of transcript levels of the indicated genes in human HSC/MFs exposed for the indicated time points to 100ng/ml human recombinant SerpinB3 (SB3). Data are expressed as means ± SEM of three independent experiments (*p< 0.05 or **p< 0.01 vs control values).

**Supplementary Figure S 3.**  Morphological comparison between liver specimens from TG-SB3 mice and related wild type mice. Immuno-histochemistry analysis has been performed to investigate hepatocyte expression of murine albumin, α-fetoprotein and cytokeratin 18 (CK-18). Positive stain for cytokeratin 7 (CK-7) was limited to cells of bile ducts or to rare hepatic progenitor cells around portal tracts. Staining of hepatocyte glycogen stores was performed using the standard PAS (periodic acid of Schiff) technique. Magnification as indicated (20x or 40 x).

**Supplementary Figure S 1.**

**Control**

**SerpinB3**

**
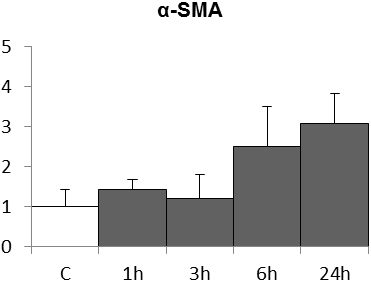

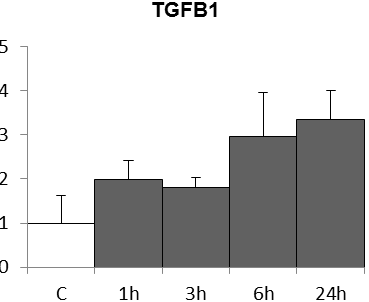
**

*

*

**2 ^–ΔΔCt^**

**2 ^–ΔΔCt^**

*

*

**
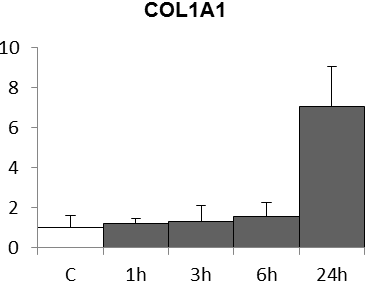

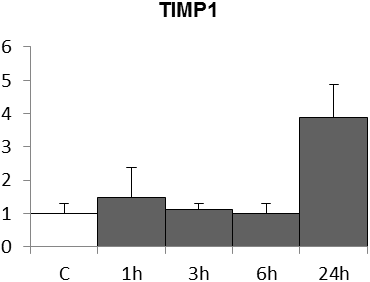
**

*

**2 ^–ΔΔCt^**

**2 ^–ΔΔCt^**

*

**
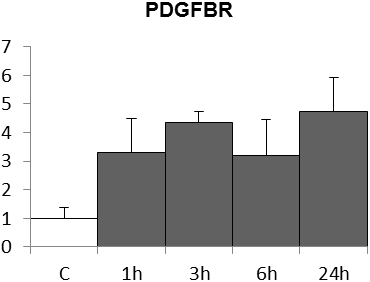

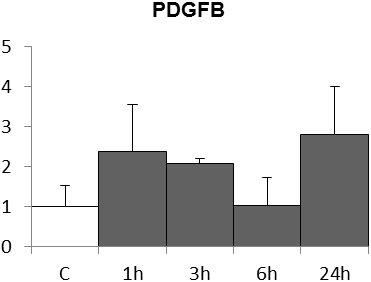
**

*

**2 ^–ΔΔCt^**

**2 ^–ΔΔCt^**

*

*

*

*

*

*

**
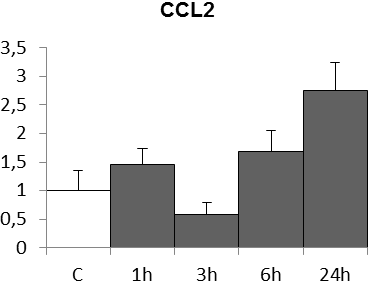

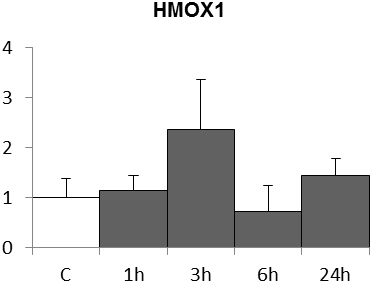
**

*

*

*

**2 ^–ΔΔCt^**

**2 ^–ΔΔCt^**

*

**Supplementary Figure S 2**

**a**

**Control**

**SerpinB3**


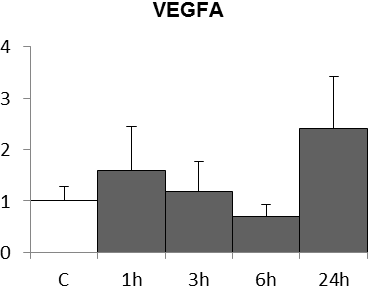


**2 ^–ΔΔCt^**

*

**b**

**c**


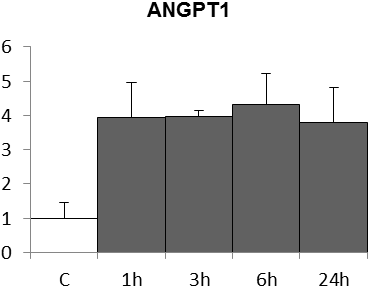

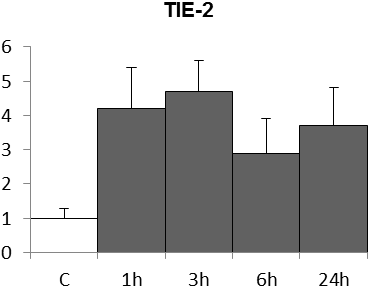


*

*

*

*

*

**2 ^–ΔΔCt^**

**2 ^–ΔΔCt^**

*

*

*

**Supplementary Figure S 3**

**TG-SB3 mice**

**WT mice**


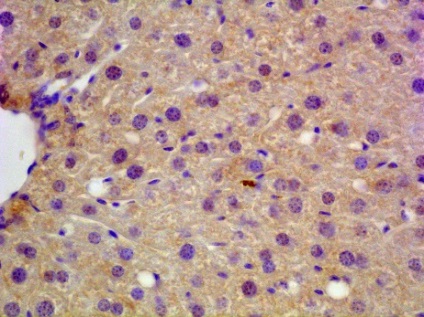

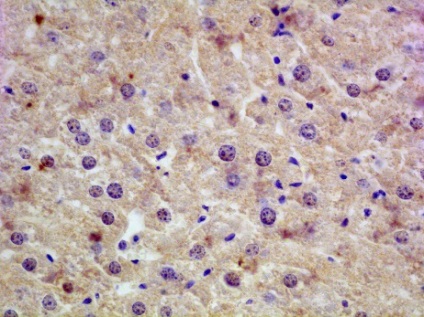


**α-FP**

**Albumin**

**CK-18**


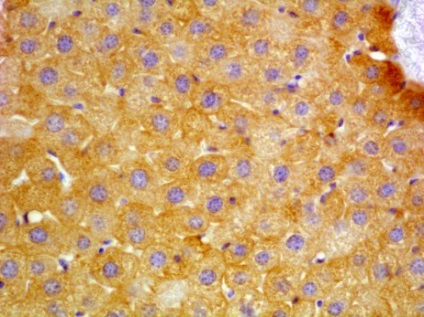

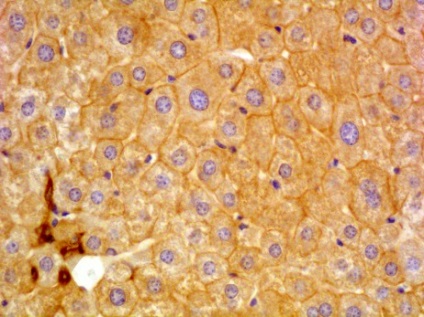

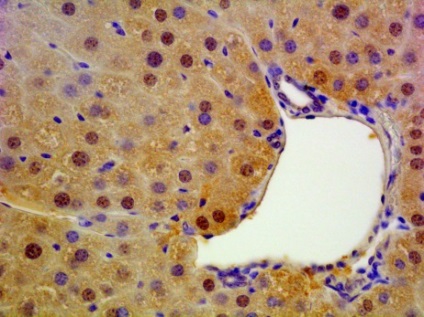

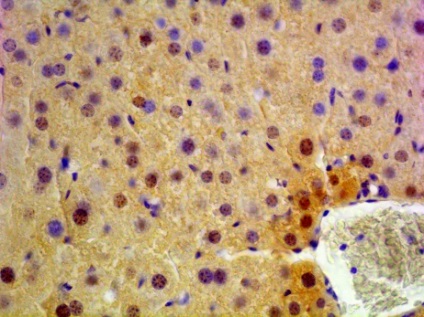

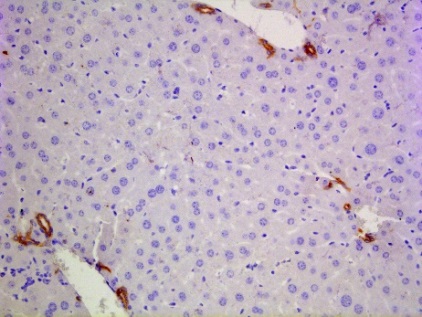

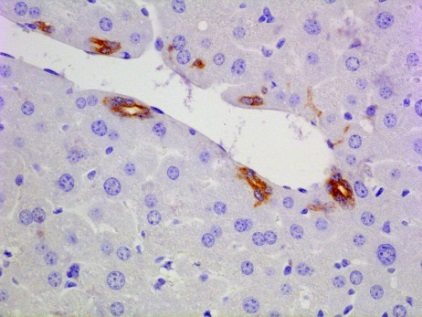

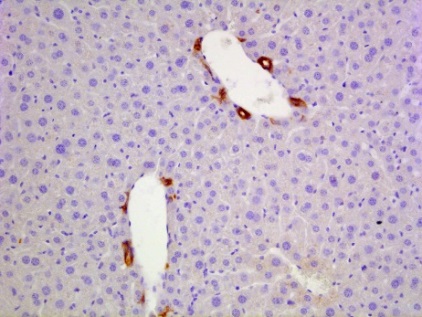

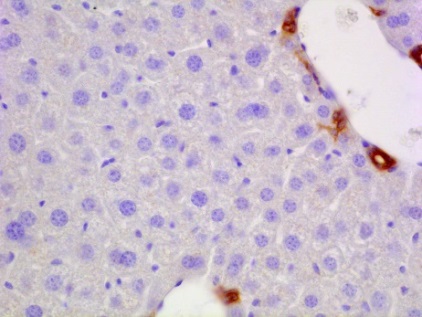


**CK-7**

**20 x**

**40 x**

**TG-SB3 mice**

**WT mice**


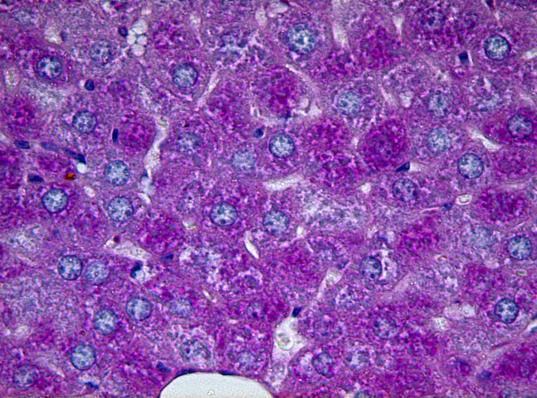

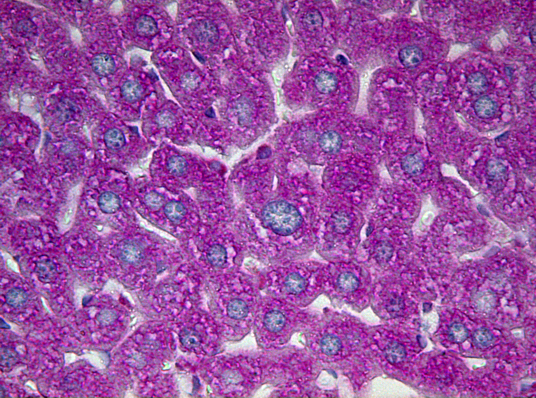


**40 x**

**40 x**

**Glycogen (PAS)**
